# Small Intestinal Intussusception Due to Complicated Giant Jejunal Diverticulosis

**DOI:** 10.3390/medicina57020116

**Published:** 2021-01-28

**Authors:** Ewa Grudzińska, Sławomir Mrowiec, Joanna Pilch-Kowalczyk, Monika Ciupińska, Katarzyna Kusnierz

**Affiliations:** 1 Department of Gastrointestinal Surgery, Medical University of Silesia, 40-752 Katowice, Poland; ewa.grudzinska@sum.edu.pl (E.G.); kkusnierzchir@gmail.com (K.K.); 2 Department of Radiology, Medical University of Silesia, 40-752 Katowice, Poland; jpk@alteris.pl; 3 Department of Pathomorphology and Molecular Diagnostics, Medical University of Silesia, 40-752 Katowice, Poland; monanet@poczta.onet.pl

**Keywords:** abdominal surgery, intussusception, diverticulosis, jejunum, lipoma, visceral surgery, malnutrition

## Abstract

*Background*: Jejunal diverticulosis and jejunal lipomatosis are uncommon conditions. Usually asymptomatic, they may cause severe complications in some cases. Intussusception is unusual in adults, but when diagnosed swiftly it can be treated surgically, usually with good outcome. *Case presentation*: We present a 60-year-old female patient with a history of chronic malnutrition and anemia, complaining of acute abdominal pain, vomiting and diarrhea. Contrast-enhanced abdominal computed tomography (CT) showed intussusception, multiple giant jejunal diverticula and multiple lipomas. The patient underwent urgent surgery, but radical treatment was not possible due to the extent of the diseases. One month later, another surgery was needed due to ileostomy obstruction caused by lipomas. The patient’s condition deteriorated due to malnutrition and concomitant metabolic disorders, which eventually led to her demise. *Conclusions*: Radical treatment is not always possible in an extensive jejunal disease. Prolonged malnutrition impairs postoperative healing, and therefore surgical or nutritional treatment should be considered in jejunal diverticulosis before the onset of severe complications requiring urgent surgical intervention.

## 1. Introduction

Jejunal diverticulosis is a rare condition affecting 0.2–4.5% of the population [1]. Usually asymptomatic, it is found accidentally during radiological investigation. In some patients, however, the disease manifests with non-specific symptoms like abdominal pain, chronic diarrhea, malabsorption or nausea [1]. Malabsorption is caused by bacterial overgrowth associated with intestinal stasis [1]. Diverticulosis can also lead to severe complications such as bleeding and perforation [2]. Computed tomography (CT) scan or magnetic resonance imaging are used for diagnosis as well as exploratory laparotomy [2]. In patients with symptoms, conservative treatment with antibiotics can be administered in order to reduce bacterial overgrowth. Surgery, the only definitive intervention, has to be considered individually.

Intussusception is a rare cause of abdominal pain in adults, best diagnosed in a CT [3]. Usually, a neoplasm is the leading point and surgery is the treatment of choice [4].

We hereby present a case of giant jejunal diverticulosis with concomitant multiple lipomas, which caused intussusception and eventually led to the patient’s death. This highlights the challenge met by a surgeon when a rare and extensive small intestine disease requires urgent surgery in a malnourished patient.

## 2. Case Presentation

**Figure 1:**
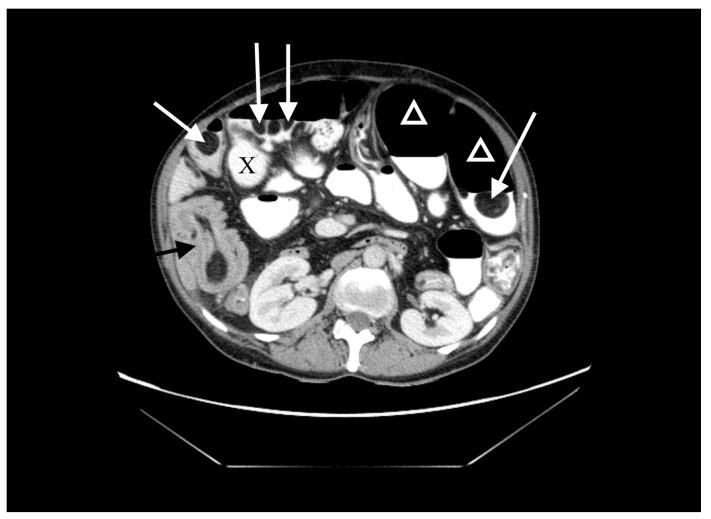
Contrast-enhanced computed tomography (CT) image of the abdomen, axial section. Multiple jejunal lipomas (white arrows), distended jejunum (triangles), one of the diverticula (X) and intussuscepted jejunum with a lipoma as the leading point (black arrow).

**Figure 2:**
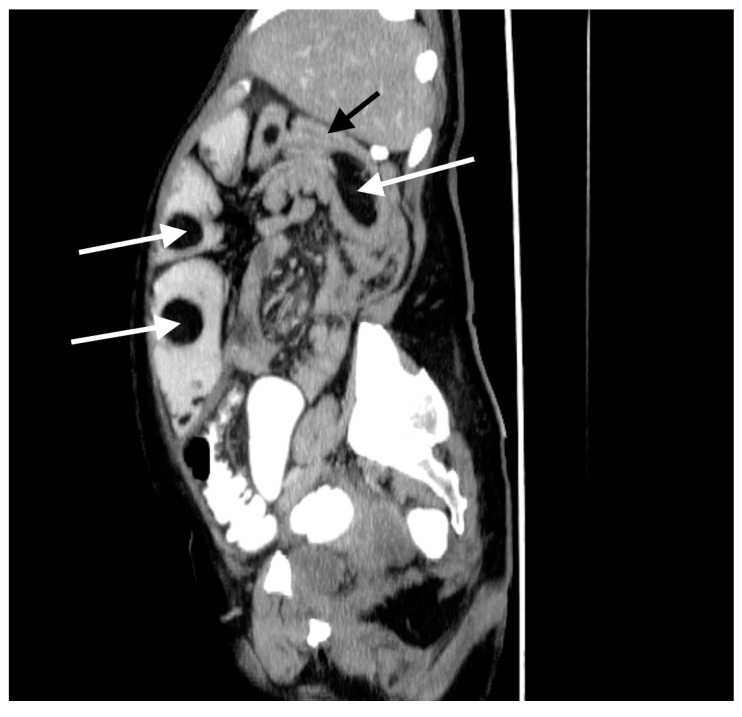
Contrast-enhanced CT image of the abdomen and pelvis, sagittal section. Multiple jejunal lipomas (white arrows) and intussuscepted jejunum with a lipoma as the leading point (black arrow).

**Figure 3:**
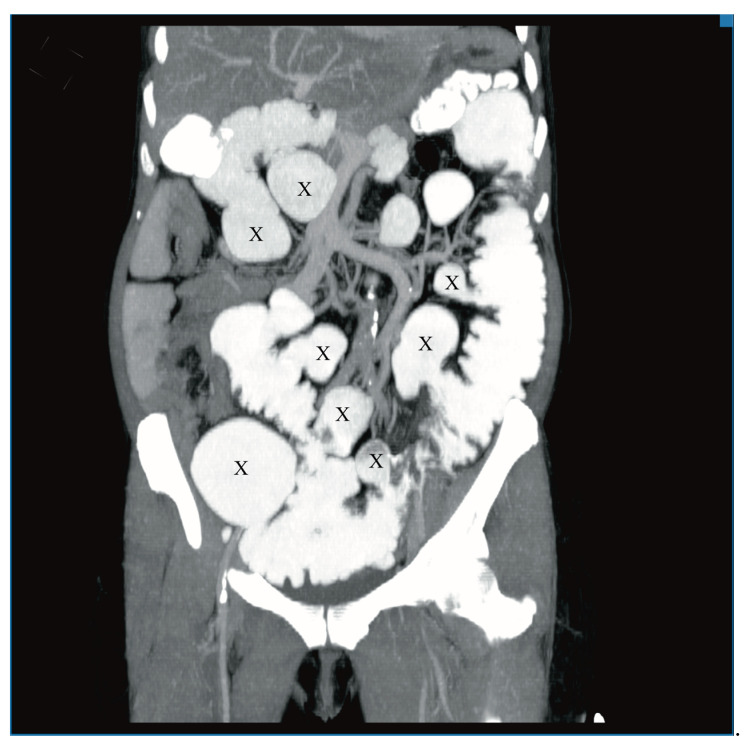
Contrast-enhanced CT image of the abdomen and pelvis, frontal section. Multiple giant diverticula (X).

A 60-year-old woman was admitted to the emergency department complaining of acute abdominal pain, vomiting and diarrhea. Her medical history included appendectomy in childhood, hysterectomy, hepatic steatosis, chronic kidney disease (CKD) and chronic malnutrition with iron deficiency. She has also been treated for indeterminate inflammatory bowel disease (she reported taking sulfasalazine, 1.0 g three times daily) and had a twenty-year history of recurrent abdominal pain accompanied by a feeling of fullness and abdominal distension. Twenty-six years prior to admission, she underwent surgery for ileus and a large polyp in the ileum was locally resected. The patient did not provide documentation from her previous hospitalizations in other wards. Physical examination revealed absence of bowel sounds and generalized abdominal tenderness. Laboratory tests showed mild anemia and low serum protein level. Abdominal CT scan demonstrated small bowel distension (Figure 1) with ileo-ileal intussusception caused by one of multiple pedunculated lipomatous polyps arising from the mesenteric border of the jejunum (Figure 1 and Figure 2). Multiple giant diverticula were also visualized (Figure 1 and Figure 3).

**Figure 4:**
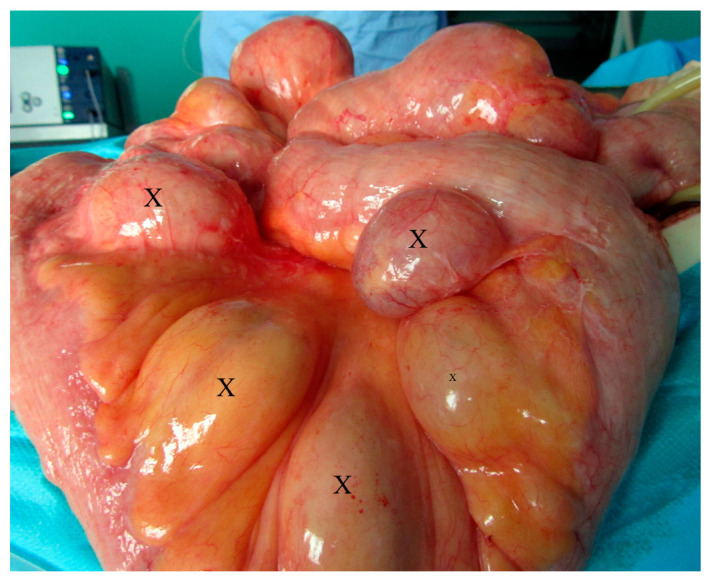
Intraoperative view of the giant jejunal diverticula (X).

**Figure 5:**
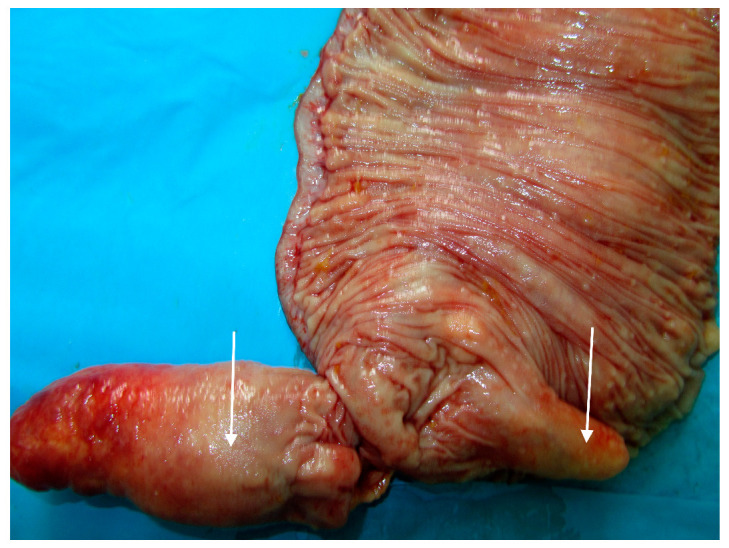
Resected specimen of the small intestine, longitudinally cut-lipomas (white arrows).

The patient underwent urgent laparotomy. Circa one meter of small intestine with multiple irregular polyps and diverticula (Figure 4 and Figure 5) was resected and ileostomy was performed.

**Figure 6:**
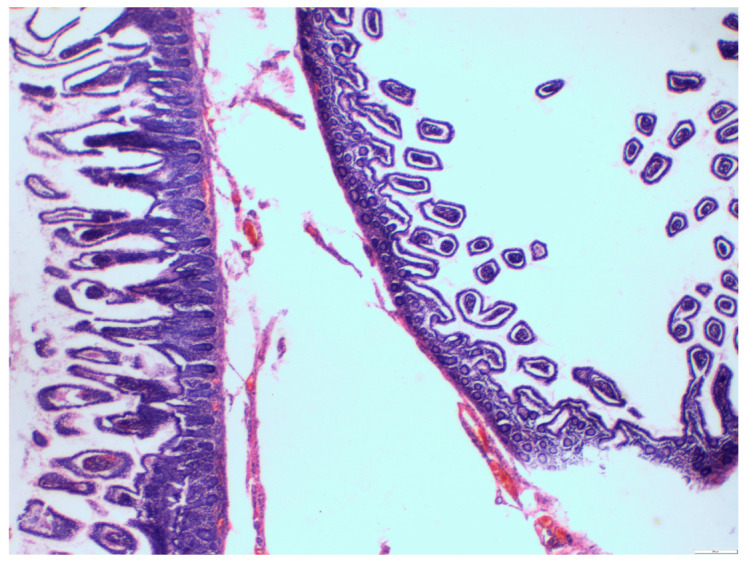
A histological view, hematoxylin and eosin, ×20. Small intestine mucosa on the left) and submucosal pseudodiverticulum (on the right).

**Figure 7:**
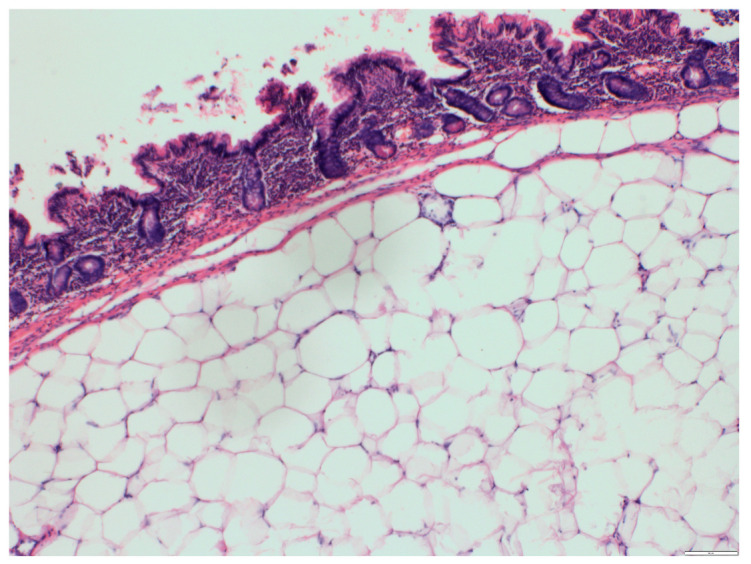
A histological view, hematoxylin and eosin, ×40. Small intestine. Submucosal lipoma composed of mature adipose tissue.

The pathological report described large pseudodiverticula and multiple submucosal lipomas composed of mature adipose tissue (Figure 6 and Figure 7).

When the patient was in stable condition, she was discharged home at her own request. Soon, she was readmitted to the hospital and treated in the Internal Diseases Department due to acute exacerbation of CKD.

One month after the first surgery, three pedunculated polyps obstructed the ileostomy. The polyps were surgically removed through the ileostomy; however, malnutrition caused by diverticulosis and CKD exacerbation led to multiorgan failure and eventually to the patient’s death.

## 3. Discussion

The case not only shows two extremely rare conditions existing in one patient, it also presents the challenge of treating an extensive disease of small intestine. Although in asymptomatic cases, small intestine diverticulosis can remain untreated, if symptoms are present, therapy should be taken into consideration [5,6]. Antibiotics are administered for malabsorption caused by bacterial overgrowth [5] and endoscopy is employed in case of bleeding or if resection of duodenal diverticula is needed [1]. Surgical removal of the affected intestine is the only definitive treatment, recommended in case of complications [1,2,7]. The patient had never received any treatment for jejunal diverticulosis, which may have been related to her reluctance to comply with medical advice.

Considering the long history of abdominal pain and malnutrition in the patient, it is possible that her disease had a genetic background. Genetic disorders manifesting with multiple lipomas have been reported previously. Germline mutations causing hamartoma tumor syndrome can present with intestinal lipomatosis already in childhood [8]. Autosomal dominant inheritance has also been implied [9]. It is suggested that in patients with lipomatosis, concomitant diverticula are caused by the tendency for abnormal fat infiltration, which weakens the intestinal wall [9,10]. In this patient, there was no family history of lipomatosis and she has never received any genetic counselling. During her stay in our ward, genetic testing was rendered secondary due to the urgency of treatment. The diverticula and lipomas were irregular, with no positional relationship. The pathomorphology report described lipomas composed of mature adipose tissue and pseudodiverticula.

In this patient, diverticulosis caused malnutrition but the indication for urgent surgery was small intestine intussusception, which, in adults, is a rare cause of obstruction. It is mainly caused by benign tumors and has good prognosis if the diagnosis is swift and urgent surgery is performed [4,11]. In this case, the diagnosis was obvious after CT scan. However, the benign lipoma causing intussusception was only one of multiple jejunal lipomas and definitive treatment was deemed impossible. This was the case in all three surgeries the patient underwent, and all her surgeries were urgent. It remains an open question whether previous nutritional therapy, antibiotics and a scheduled surgery with extensive small intestine resection and risk of lifelong parenteral nutrition could have been beneficial for the patient.

## 4. Conclusions

The case not only illustrates a rare medical condition but also highlights the challenges of treating an extensive jejunal disease when a radical procedure is impossible. Diseases affecting a large portion of small intestine require careful evaluation of the possible surgical treatment before the onset of severe complications and the patients should always receive nutritional advice.

## Data Availability

Data available on request: the data presented in this study are available on request from the corresponding author. The data are not publicly available due to the patient’s privacy.
